# Evaluation of Three Protein-Extraction Methods for Proteome Analysis of Maize Leaf Midrib, a Compound Tissue Rich in Sclerenchyma Cells

**DOI:** 10.3389/fpls.2016.00856

**Published:** 2016-06-14

**Authors:** Ning Wang, Xiaolin Wu, Lixia Ku, Yanhui Chen, Wei Wang

**Affiliations:** State Key Laboratory of Wheat and Maize Crop Science, Collaborative Innovation Center of Henan Grain Crops, Henan Agricultural UniversityZhengzhou, China

**Keywords:** 2DE-based proteomics, differential abundant proteins, maize leaf midrib, protein extraction, sclerenchyma cell

## Abstract

Leaf morphology is closely related to the growth and development of maize (*Zea mays* L.) plants and final kernel production. As an important part of the maize leaf, the midrib holds leaf blades in the aerial position for maximum sunlight capture. Leaf midribs of adult plants contain substantial sclerenchyma cells with heavily thickened and lignified secondary walls and have a high amount of phenolics, making protein extraction and proteome analysis difficult in leaf midrib tissue. In the present study, three protein-extraction methods that are commonly used in plant proteomics, i.e., phenol extraction, TCA/acetone extraction, and TCA/acetone/phenol extraction, were qualitatively and quantitatively evaluated based on 2DE maps and MS/MS analysis using the midribs of the 10th newly expanded leaves of maize plants. Microscopy revealed the existence of substantial amounts of sclerenchyma underneath maize midrib epidermises (particularly abaxial epidermises). The spot-number order obtained via 2DE mapping was as follows: phenol extraction (655) > TCA/acetone extraction (589) > TCA/acetone/phenol extraction (545). MS/MS analysis identified a total of 17 spots that exhibited 2-fold changes in abundance among the three methods (using phenol extraction as a control). Sixteen of the proteins identified were hydrophilic, with GRAVY values ranging from -0.026 to -0.487. For all three methods, we were able to obtain high-quality protein samples and good 2DE maps for the maize leaf midrib. However, phenol extraction produced a better 2DE map with greater resolution between spots, and TCA/acetone extraction produced higher protein yields. Thus, this paper includes a discussion regarding the possible reasons for differential protein extraction among the three methods. This study provides useful information that can be used to select suitable protein extraction methods for the proteome analysis of recalcitrant plant tissues that are rich in sclerenchyma cells.

## Introduction

The continued steady increase in the world's population, particularly in developing countries, presents a major challenge with regard to food security worldwide. Maize (*Zea mays* L.) is a globally important C4 cereal crop, as evidenced by its production of approximately 1021 million tons in 2014 (FAO). Maize is predicted to become the first-ranked crop globally by 2020, thus making maize production critical to sustainable food security worldwide. Hybrid seeds and production technologies have been used extensively to promote maize production as a means of coping with a changing climate and a growing population (Gong et al., 2015). Increasing planting density is a practical means of obtaining higher maize yields in areas with limited arable land (Shafi et al., 2012). Therefore, there is great interest among maize researchers and breeders with regard to density resistance characteristics and plant architectures, particularly leaf morphology.

The leaf is the main photosynthetic organ of plants and plays an important role in dry matter accumulation. Leaf morphology (e.g., leaf area index, leaf thickness and leaf angle) affects photosynthesis efficiency (Liu et al., 2003; Oguchi and Hikosaka, 2003; Posada et al., 2012) and largely determines the final yield (Chen et al., 2013). The maize leaf develops a strong midrib to situate the blade for maximum sunlight capture. The midrib contains two types of supporting tissues: vascular tissue, including xylem and phloem, and mechanical tissue, including sclerenchyma and collenchyma. The vascular system is mainly composed of xylem and phloem, which distributes and interconnects both root and shoot systems. The vascular system within leaf midribs is responsible for mechanical support as well as the long-distance and two-way transport of water (Sano et al., 2005; Brodersen and McElrone, 2013, inorganic ions Miller et al., 2001), and photosynthetic products (Lalonde et al., 2003), including proteins and nucleic acids (Omid et al., 2008; Kehr, 2009). Collenchyma provides flexible support that supplements the function of turgid parenchyma. Recently, we speculated that collenchyma might contribute to the mechanical properties of maize leaf midribs (Wang et al., 2015). Sclerenchyma or collenchyma generally forms continuous layers beneath the epidermises of midrib regions in sugarcane leaves (Joarder et al., 2010). Sclerenchyma also provides tensile or compression strength in other parts of the plant, such as the vascular tissues of stems and roots and the bundle sheaths of leaves. The cells of both types of supporting tissues have thick walls with varied degrees of lignification. Cell wall lignification is a complex process involved in the deposition of lignin, which contains various monomeric phenolics within the extracellular polysaccharide matrix (Barcelo, 1997; Wang et al., 2013). Due to the substantial existence of thickened cell walls, high amounts of non-protein substances, and low amounts of proteins, it is very difficult to extract high-quality proteins from maize leaf midribs for 2DE analysis.

A successful 2DE-based proteomic analysis is highly dependent on high-quality gel maps, which depend on protein sample quality. In this regard, a number of protein extraction methods have been established to study plant proteomics (e.g., Damerval et al., 1986; Hurkman and Tanaka, 1986; Wang et al., 2003; Carpentier et al., 2005; Wu et al., 2014a). In particular, TCA/acetone extraction and phenol extraction are commonly used for removing non-protein substances in plant crude extracts. In most cases, TCA/acetone or phenol-based methods are modified to make them suitable for various recalcitrant plant tissues (e.g., Mijnsbrugge et al., 2000; Carpentier et al., 2005; Wang et al., 2007). Given the complexity of the maize midrib in structure and composition, protein extraction methods suitable for gel-based proteomic analysis must be evaluated and optimized. In the present study, three protein-extraction methods commonly used in plant proteomics, phenol extraction, TCA/acetone extraction, and TCA/acetone/phenol extraction, were qualitatively and quantitatively evaluated based on 2DE maps and MS/MS analysis. Our results provide useful information for selecting suitable protein extraction methods for the proteome analysis of recalcitrant plant tissues rich in sclerenchyma cells.

## Materials and methods

### Plant materials

Maize inbred 87-1 was grown in the field at an experimental farm of Henan Agriculture University in Zhengzhou, China. The climate of Zhengzhou is characterized as temperate continental monsoon (average annual temperature, 14.3°C; total amount of annual precipitation, 640.9 mm). The type of soil in the experimental farm was yellow loam. The maize field was kept adequately moist by occasionally irrigating the growing region.

Adult plants of maize inbred 87-1 contain 18 or 19 leaves (5 above the ear) and can grow to a height of 175 cm. Leaf position was determined by counting the total numbers of leaves that had visible collars at their blade bases. When the 10th leaves had fully expanded, the selected plants were transferred to the laboratory with their roots in appropriate soil. The midribs of the 10th newly expanded leaves were manually separated from the surrounding mesophyll and used for microscopic and proteomic analysis.

### Microscopic observation

**Figure 1:**
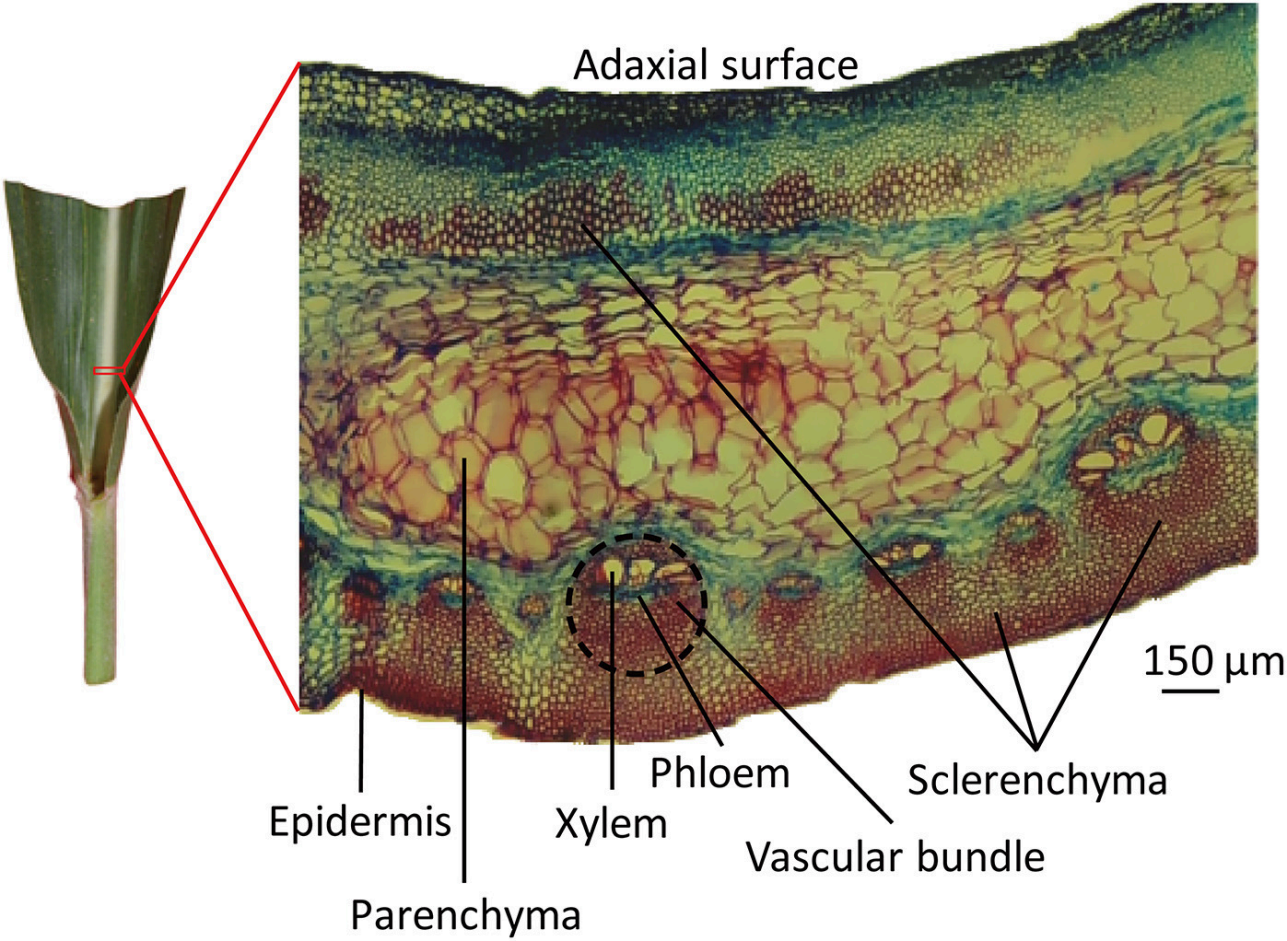
**The microscopic structure of leaf midrib of maize**. An approximately 5 mm × 5 mm piece of leaf midrib was excised via razor, at a point 2 cm from the leaf lamina joint of the midrib along the longitudinal direction of the blade. Transverse sections of leaf midribs are shown on the right. Tissue types are labeled with black lines. The vascular bundle area is circled with a black dotted line.

Using a razor, an approximately 5 mm × 5 mm piece of leaf midrib was excised at a point 2 cm from the leaf lamina joint of the midrib along the longitudinal direction of the blade (Figure 1). The midrib pieces were immediately fixed in FAA solution (10% formaldehyde, 10% acetic acid, and 45% ethanol) for 72 h and dehydrated in an ethanol series (30, 50, 75, 85, 95, and 100%). Safranin (Catalog S2255, Sigma) was added during the dehydration step to mark the tissue for orientation when sectioning later. The dehydrated tissue pieces were incubated in 3 ml xylene for 6 h. The xylene was then removed and replaced with 3 ml liquid paraffin, and the tissue pieces were incubated at 60°C for 30 min. The embedded samples were transferred to a mode (pre-filled with liquid paraffin) and cooled. A rotary microtome (Leica RM2235, Wetzlar, Germany) was used to make 5-μm thick tissue specimens. The sections were placed on slides, using Haupts A solution as an adhesive, and floated on 3% formalin at 48°C. Once the paraffin ribbons of the sections had fully expanded and were wrinkle-free, they were transferred to a 40°C environment for drying. The dried slides were deparaffinized via two changes of xylene, each occurring over 15 min. The sections were stained using Johansen's Safranin/Fast Green protocols (Johansen, 1940). The deparaffinized slides were placed in 1% aqueous safranin for 15 min and thoroughly washed in distilled water to remove excess dye. Then, the slides were counterstained in 0.5% Fast Green FCF in 95% ethanol for 30 s and thoroughly washed in 95% ethanol to remove excess dye. The stained slides were washed successively with 50% xylene in ethanol and with 100% xylene for 5 s and dried overnight. A cover-glass was added to the slide with balsam neutral (Solarbio) for permanent slide preparation. The slides were observed using a Phoenix PH50 microscope (Phoenix, China) with transmitted light. The stained sections were photographed using a digital camera attached to the microscope and recorded using the ToupView x86 software.

### Protein extraction

**Figure 2:**
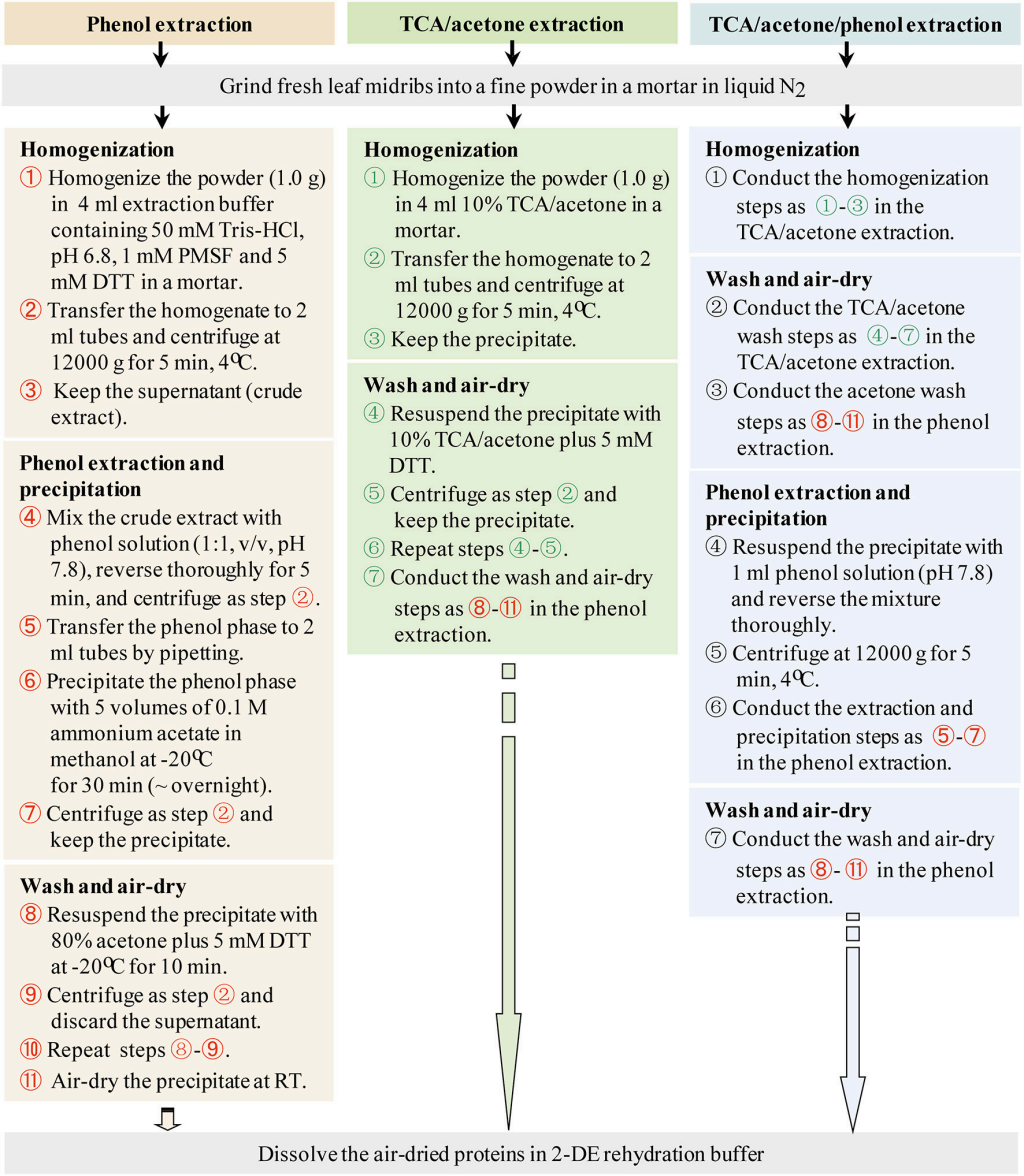
**Working flows of the three protein-extraction methods**.

Three commonly used protein extraction methods, phenol extraction, TCA/acetone extraction, and TCA/acetone/phenol extraction, were evaluated using maize leaf midribs (Figure 2). Each evaluation involved three independent biological experiments. Fresh leaf midribs were grinded using a mortar and pestle in liquid N_2_ until a fine powder was obtained. Both the 10% TCA/acetone and 80% acetone solutions used in the following steps contained 5 mM DTT.

#### Phenol extraction

The midrib powder (1.0 g) was homogenized using a mortar and pestle in 4 ml of pH 6.8 buffer containing 50 mM Tris-HCl, 5 mM DTT, and 1 mM PMSF. The homogenate was transferred into 2-ml Eppendorf tubes and centrifuged at 12,000 *g* and 4°C for 5 min. The supernatant (crude extract) was transferred to new tubes. Equal volumes of buffered phenol solution (pH 7.8) were added to the tubes and phenol extraction was performed using a methodology adapted from Wu et al. (2014a). The mixtures were thoroughly reversed for 5 min. Phase separation was achieved by centrifugation as above. The organic phase was transferred to 2-ml tubes, precipitated using 5 volumes of 0.1 M ammonium acetate in methanol at −20°C for 30 min (~overnight), and centrifuged as above. The precipitate was washed twice with 80% cold acetone. For each wash, 1.5 ml of 80% cold acetone was added, and the precipitate was vortexed thoroughly at −20°C for 10 min and centrifuged as above.

#### TCA/acetone extraction

The protocol described by Damerval et al. (1986) was followed with a slight modification. The midrib powder (1.0 g) was homogenized in 4 ml of 10% TCA/acetone containing 5 mM DTT using a mortar. The homogenate was transferred to 2-ml Eppendorf tubes and centrifuged at 12,000 *g* at 4°C for 5 min. The precipitate was washed with 10% TCA/acetone, and the mixture was thoroughly vortexed and centrifuged as above. Each 10% TCA/acetone wash was repeated once. The resulting precipitate was washed using 80% cold acetone following the washing procedure used for phenol extraction.

#### TCA/acetone/phenol extraction

It was performed using a methodology adapted from Wu et al. (2014a). The midrib powder (1.0 g) was homogenized in 4 ml of 10% TCA/acetone containing 5 mM DTT using a mortar and pestle. The homogenate was then transferred to 2-ml Eppendorf tubes and the mixture was centrifuged at 12,000 *g* at 4°C for 5 min. The precipitate was collected and washed with 10% TCA/acetone and 80% acetone following the washing procedure used for TCA/acetone extraction. The precipitate was resuspended in 1 ml of phenol solution (pH 7.8), thoroughly reversed for 5 min, and centrifuged as above. The supernatant was precipitated using 5 volumes of 0.1 M ammonium acetate in methanol and washed as described above in “phenol extraction.”

In addition, Tris-HCl extraction followed by acetone precipitation or chloroform-methanol precipitation was performed in preliminary experiments as a control extraction. The midrib powder (1.0 g) was homogenized in a mortar and pestle in 4 ml of pH 6.8 buffer containing 50 mM Tris-HCl, 5 mM DTT, and 1 mM PMSF. The homogenate was transferred into 2 ml Eppendorf tubes and centrifuged at 12,000 g and 4°C for 5 min. The supernatant (crude extract) was precipitated by four volume of cold acetone (Zivy et al., 1983) or chloroform-methanol (Wessel and Flügge, 1984).

The final protein precipitates from mentioned-above extractions were air-dried for 10 min and then dissolved in the 2DE rehydration buffer (8 M urea, 2 M thiourea, 2% CHAPS, 20 mM DTT, with or without 0.5% IPG buffer, pH 4-7). For protein assay before 2DE, protein precipitates were dissolved in a 2DE rehydration buffer without the IPG buffer to avoid its interference. Protein concentrations were determined using Bradford (1976) method with bovine serum albumin as a standard. After then, an aliquot of the IPG buffer was supplemented into protein samples to a concentration of 0.5% (v/v).

### 2DE

Before 2DE, protein samples were centrifuged to remove insoluble substances. First-dimensional IEF was performed using 11-cm pH 4-7 linear IPG strips and a PROTEAN IEF system (Bio-Rad). All IPG strips were rehydrated at 20°C overnight using 250 μl of rehydration buffer, which contained approximately 600 μg of proteins. The voltage settings for IEF were as follows: a linear increase from 0 to 250 V over 3 h, from 250 V to 4000 V over 4 h, from 4000 V to 8000 V over 4 h, and then held at 8000 V for a total of 15 kV h (20°C). For the secondary SDS-PAGE, the focused strips were equilibrated on a shaker for 15 min in a buffer containing 0.1 M Tris-HCl (pH 8.8), 2% SDS, 6 M urea, 30% glycerol, and 0.1 M DTT, followed by 15 min in the same buffer with 0.25 M iodoacetamide in place of DTT. The strips were washed with water and loaded onto 12.5% polyacrylamide gels (20 × 15 × 0.1 cm). Electrophoresis was performed in SDS-PAGE running buffer for 3 h at 15°C using constant power settings (20 mA).

After electrophoresis, gels were fixed with 40% methanol and 10% acetic acid for 30 min and then stained in 0.1% Coomassie Blue G-250 for 2-4 h and distained in 5% acetic acid. The 2DE images were digitalized with a camera (Nikon d7000, Japan), and processed using PDQUEST software (Bio-Rad, USA). The gels from phenol extraction method were taken as master gels. Test settings are as follows: sensitivity: 6.86; size scale: 9; min peak: 1319.

### MS/MS

Spots exhibiting 2-fold abundance changes between any two of the three protocols were digested and analyzed using a MALDI-TOF-TOF analyzer (AB SCIEX TOF/TOF-5800, AB SCIEX, USA). Spots were excised from the gels, reduced (10 mM DTT), alkylated (50 mM iodoacetic acid), and then digested with 10 mg/ml trypsin for 16 h at 37°C in 50 mM ammonium bicarbonate. The supernatants were vacuum-dried and dissolved in 10 μl of 0.1% trifluoroacetic acid, after which 0.5 μl was added to a matrix consisting of 0.5 μl of 5 mg/ml 2,5-dihydroxybenzoic acid in water and acetonitrile (2:1). Mass spectra were acquired in the positive ion mode and automatically submitted to Mascot 2.2 (http://www.matrixscience.com) for identification against NCBInr 20150823 database (70,861,097 sequences, 25,660,401,164 residues, http://www.ncbi.nlm.nih.gov/). The taxonomy was viridiplantae (green plants) (3,136,015 sequences). The search parameters were as follows: type of search: MALDI-TOF ion search; enzyme: trypsin; variable modifications: acetyl (protein N-term), deamidated (NQ), dioxidation (W) and oxidation (M); fixed modifications: carbamidomethyl (C); mass values: monoisotopic; protein mass: unrestricted; peptide mass tolerance: ±20 ppm; fragment mass tolerance: ±0.2 Da; max missed cleavages: 1; instrument type: MALDI-TOF-TOF. As defined by Mascot probability analysis, only significant scores greater than “identity” were considered for assigning protein identity. All of positive protein identification scores were significant (*p* < 0.05, score > 52).

### Bioinformatics analysis

Grand average of hydropathicity (GRAVY) values was calculated for the proteins that were identified using ProtParam (http://web.expasy.org/protparam). Subcellular localization, molecular function, and biological process were the annotations considered via UniProtKB (http://www.uniprot.org/uniprot/). In some cases, subcellular localizations of proteins without annotations in UniProtKB were predicted using SubLoc v1.0 (http://www.bioinfo.tsinghua.edu.cn/SubLoc/eu_predict.htm). The molecular functions or biological processes of uncharacterized proteins were searched by BLAST using UniProtKB accessions (http://www.uniprot.org/blast/).

## Results

### Microscopy of maize leaf midrib sections

Transverse sections of fresh midrib pieces of 10th newly expanded leaves were stained using Safranin/Fast Green protocol and observed under a microscope. In the midribs, various types of tissues, i.e., vascular bundles, parenchyma, sclerenchyma and collenchyma, were found (Figure 1). Epidermises formed the outermost protective covering of the midrib. Vascular bundles were close to the abaxial side of leaf midribs, where vascular tissues included xylem, phloem, and sclerenchyma in distinct regions. There were two types of vascular bundles: the larger consisted of 3–6 xylem vessels with diameters of 30–70 μm, and the smaller contained xylem vessels with diameters of 10–40 μm. Moreover, three or four small vascular bundles existed between the larger vascular bundles. The sizes of sclerenchyma cells were similar to the sizes of small vessels. Parenchyma cells were sandwiched between vascular bundles and adaxial sclerenchyma and occupied substantial areas. Parenchyma cells were distinguishable from cells of vascular tissues given the former's large cell sizes, which ranged from 80 to 150 μm.

Phloem and xylem were spatially separated, with phloem stained green, suggesting a lack of secondary walls. Xylem cell walls were stained deep red, indicating a high degree of vessels lignification. Continuous sclerenchyma cells divided the abaxial epidermis from vascular bundles and gave an intense red color, indicating the presence of lignin within the thickened cell walls. Groups of sclerenchyma cells with intense red walls were found beneath the adaxial epidermis. In addition, although it is commonly recognized that maize is a C4 plant with Kranz structure, this structure was not found in the sections of vascular bundles in maize leaf midribs in the present study.

### Proteomic analysis of maize leaf midribs

In preliminary experiments, Tris-HCl extraction followed by acetone precipitation or chloroform-methanol precipitation did not produce good 2DE images, compared to phenol extraction and TCA/acetone precipitation (Supplementary Image 1), demonstrating that maize midribs are a particularly recalcitrant tissue for proteomics analysis. Thus, our evaluation focused on the three common extraction methods in plant proteomics, i.e., phenol extraction, TCA/acetone extraction, and TCA/acetone/phenol extraction (Figure 2).

**Figure 3:**
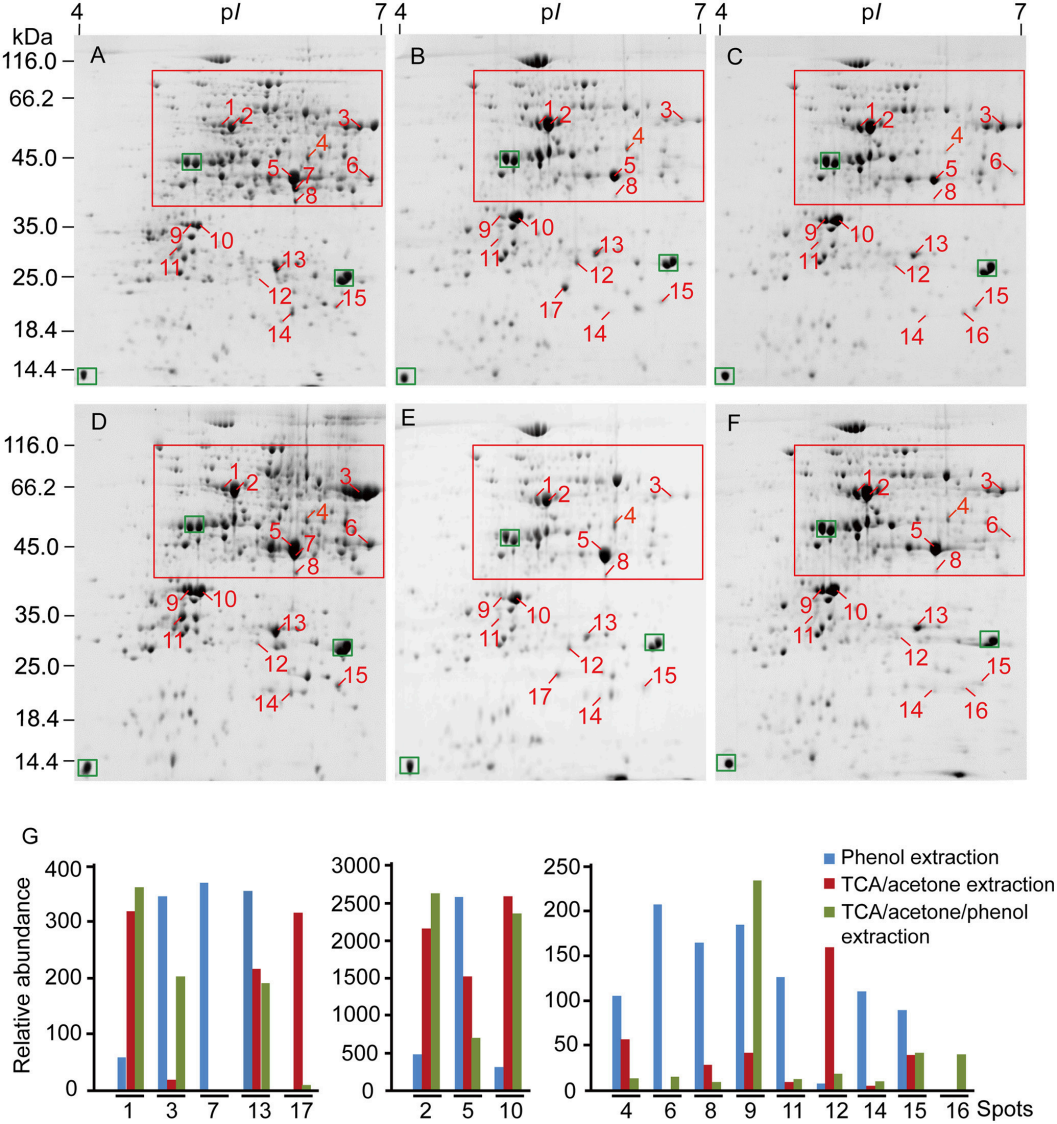
**2DE analysis of differentially extracted proteins from maize leaf midribs among the three protein-extraction methods. (A–C)** Correspond to phenol extraction, TCA/acetone extraction and TCA/acetone/phenol extraction, respectively. **(D–F)** Constitute a group of replicates of **(A–C)**. About 600 μg of proteins was loaded on a pH 4-7, non-linear gel strip for IEF and were then separated on a 12.5% SDS-PAGE before Coomassie Brilliant Blue G250 staining. Differentially extracted proteins were numbered in 2DE images. The green boxes indicated landmark proteins. The red boxes indicated the regions with more differential spots. **(G)** abundance comparison of differentially extracted protein spots. Spot volumes were normalized and determined using PDQuest, representing the mean of the three extraction methods.

Protein yield extracted from leaf midribs varied with the methods used (Table 1). Higher protein yields were extracted via TCA/acetone extraction compared with the other two methods. However, phenol extraction resulted in higher numbers of spots in 2DE images compared with TCA/acetone-based extractions, particularly at the acidic ends and high molecular mass regions indicated by red boxes (Figure 3).

**Table 1:** **Comparison of the three protein-extraction methods using maize leaf midribs**.

**Method**	**Protein yield (mg/g dry weight)**	**Spot number A^a^**	**Time (h)^b^**	**Match to all (correlation of coefficient %)**	**Match rate (%)^c^**	**Spot in red box^d^**
Phenol extraction	1.64 ± 0.01	655 ± 9	1.5	285 (0.843)	Master gel	239 ± 13
TCA/acetone extraction	3.08 ± 0.21	589 ± 25	1.0	208 (0.830)	62 ± 6	109 ± 18
TCA/acetone/phenol extraction	1.77 ± 0.04	545 ± 6	2.5	215 (0.831)	53 ± 3	133 ± 11

a *Spot number in 2DE images detected using auto spot detection module of PDQUEST software (Bio-Rad)*.

b *Time consumed by each protein extraction method*.

c *Match rate calculated taking gel image of phenol extraction as the master*.

d *Spot numbers in red boxes of Figure 3*.

Efficient protein separation and good spot focusing were observed in the 2DE images obtained for all three extraction methods (Figure 3). High-quality protein charge trains of high molecular mass were obtained without horizontal and vertical streaking problems. Phenol extraction produced more even spot distribution across the near-neutral region (p*I* 5 to 7). Relative abundances of major high-abundance proteins (labeled with green boxes) revealed no obvious variations in the gel images of the three extractions. However, spot numbers were higher in gel images of phenol extractions compared with those of TCA/acetone-based extractions. Similar correlation coefficient values were obtained for the three extraction methods. The 2DE images obtained for TCA/acetone extraction produced higher match rates compared with those obtained for TCA/acetone/phenol extraction (Table 1). These results indicate that small amounts of proteins were lost in the following protein separation, precipitation or washing steps of the TCA/acetone/phenol extraction method.

In the present study, 17 proteins that were differentially extracted by the three extraction methods were identified via MALDI-TOF-TOF (Supplementary Table 1). Spot 7 was only detected in the phenol extraction, and spot 16 was only in the TCA/acetone/phenol extraction. The 17 differential spots were quantifiably compared based on their volumes among the three extraction methods (Figure 3). The proteins identified predominantly played roles in photosynthesis (spots 3, 9, 10, 12, 16, and 17), glycolysis (spots 5–8), transport (spots 1 and 2), and protein metabolism (spots 14 and 15).

Eight identified proteins were located within the chloroplast membrane system. In addition, six identified proteins were predicted to localize in mitochondria, and the other proteins were cytoplasmic or nuclear (Table 2).

**Table 2:** **Characterization of identified proteins among the three protein-extraction methods in maize leaf midribs**.

**Spot**	**NCBI accession**	**UniProtKB accession**	**Protein**	**GRAVY^a^**	**Subcellular localization (Identity)^b^**	**Molecular function^c^**	**Biological process^d^**
1	227786	P00827	ATP synthase subunit beta	−0.078	CF(1), Chloroplast, Membrane, Plastid, Thylakoid	ATP binding, Rotational mechanism, Proton-transporting ATP synthase activity	ATP synthesis coupled proton transport
2	227786	P00827	ATP synthase subunit beta	−0.078	CF(1), Chloroplast, Membrane, Plastid, Thylakoid	ATP binding, Rotational mechanism, Proton-transporting ATP synthase activity	ATP synthesis coupled proton transport
3	131998	P19163	RuBisCO large subunit; Flags: Precursor	−0.273	Chloroplast, Plastid	Magnesium ion binding, Monooxygenase activity, Ribulose-bisphosphate carboxylase activity	Photorespiration Reductive pentose-phosphate cycle
4	226508814	B6T9J4	Aspartate aminotransferase	−0.153	Mitochondrion (74%)	Pyridoxal phosphate binding, Transaminase activity	Biosynthetic process Cellular amino acid metabolic process
5	223975775	C0PD30	Fructose-bisphosphate aldolase	−0.175	Mitochondrion (91%)	Fructose-bisphosphate aldolase activity	Glycolytic process
6	22240	Q6LBU9	Glyceraldehyde-3-phosphate dehydrogenase	−0.066	Cytoplasm (84%)	NAD binding and NADP binding, Oxidoreductase activity	Glucose metabolic process
7	223975775	C0PD30	Fructose-bisphosphate aldolase	−0.175	Mitochondrion (91%)	Fructose-bisphosphate aldolase activity	Glycolytic process
8	223975775	C0PD30	Fructose-bisphosphate aldolase	−0.175	Mitochondrion (91%)	Fructose-bisphosphate aldolase activity	Glycolytic process
9	670412710	B6T3B2	Oxygen-evolving enhancer protein 1	−0.221	Extrinsic component of membrane, Integral component of membrane, Photosystem II oxygen evolving complex	Calcium ion binding	Photosynthesis, Photosystem II stabilization
10	670412710	B6T3B2	Oxygen-evolving enhancer protein 1	−0.221	Extrinsic component of membrane, Integral component of membrane, Photosystem II oxygen evolving complex	Calcium ion binding	Photosynthesis, Photosystem II stabilization
11	195642948	B6TV09	3-beta hydroxysteroid dehydrogenase/isomerase family protein	0.162	Mitochondrion (56%)	Coenzyme binding, Isomerase activity	Response to oxidative stress
12	413954857	K7VFU9	Chlorophyll a-b binding protein 8	−0.026	Chloroplast, Membrane, Plastid, Photosystem II, Thylakoid	Chlorophyll binding	Photosynthesis, Light harvesting, Protein-chromophore linkage
13	226505920	B4FT31	Dehydroascorbate reductase	−0.185	Cytoplasm (94%)	Glutathione dehydrogenase (ascorbate) activity	Detoxification Stress response
14	162458009	P80639	Eukaryotic translation initiation factor 5A	−0.487	Nuclear (56%)	Ribosome binding Translation elongation/initiation factor activity	Positive regulation of translational elongation and termination, Traslational frameshifting
15	226491656	B6TCE9	Peptidyl-prolyl cis-trans isomerase	−0.157	Mitochondrion (56%)	Peptidyl-prolyl cis-trans isomerase activity	Protein folding
16	194702912	B4FTU7	Cytochrome b6-f complex iron-sulfur subunit	−0.130	Integral component of membrane, Thylakoid membrane	Oxidoreductase	Electron transport, Transport
17	226530077	B4FZL4	Chlorophyll a-b binding protein 6A	−0.105	Chloroplast, Membrane, Photosystem I, Photosystem II, Plastid, Thylakoid	Chlorophyll binding, Metal ion binding	Light harvesting Protein-chromophore linkage, Photosynthesis

a *Grand average of hydropathicity (GRAVY) calculated by ProtParam (http://web.expasy.org/protparam)*.

b *Subcellular localization according to UniProtKB (http://www.uniprot.org/uniprot/). The proteins without subcellular localization annotation in UniProtKB were predicted using SubLoc v1.0 (http://www.bioinfo.tsinghua.edu.cn/SubLoc/eu_predict.htm). The numbers in the brackets refer to the identities of the predictions*.

c *Molecular function*.

d *Biological process according to annotations in UniProtKB (http://www.uniprot.org/uniprot)*.

GRAVY consists of important factors related to protein hydrophilicity. The GRAVY values for 16 of the proteins identified ranged from -0.026 to -0.487 (Table 2). The GRAVY value obtained for the 3-beta hydroxysteroid dehydrogenase/isomerase family protein (spot 11) was the only positive value obtained. The abundance of this nuclear protein differed among the three extraction methods utilized. The phenol extraction method resulted in higher amounts of 3-β-hydroxysteroid dehydrogenase/isomerase than the TCA/acetone-based methods, suggesting that phenol extraction had a higher capacity for extracting particular proteins (i.e., 3-β-hydroxysteroid dehydrogenase/isomerase).

## Discussion

### The microscopic structure of maize leaf midribs

Maize leaf midribs were characterized by large xylem vessels and massive phloem cells. The sclerenchyma was hardened by lignin and bound together to provide strength and support within the vascular system. The number of these specialized cells increases as the plant grows. Sclerenchyma is an obvious characteristic of the midrib structure of maize, a typical C4 plant (Vermerris et al., 2010).

In the present study, we observed that bundle sheath cells had transformed into sclerenchymatous bundle cover of maize leaf midrib, which was accompanied by a complete lack of Kranz structure. Microscopic observation revealed patches of sclerenchyma beneath the abaxial epidermis in the leaf midribs of sugarcane plants (Joarder et al., 2010). However, we observed here continuous sclerenchyma under the abixial epidermis and patches of sclerenchyma beneath the adixial epidermis in leaf midribs of adult maize plants. Thus, the extent of differentiation (patched or continuous) of sclerenchyma cells in leaf midribs likely varied with maize leaf age. Therefore, an increased number of sclerenchyma cells in adult plants likely results in difficulties related to protein extraction from maize leaf midribs. Substantial parenchyma makes it difficult to completely pulverize maize leaf midrib such that harsh breaking conditions are required, such as grinding in a mortar with liquid N_2_.

Plant maturity was obviously accompanied by large accumulations of secondary metabolites and particularly phenolics, which greatly interfere with plant extraction and separation. Interfering substances, including starch, lipids, and secondary metabolites, may result in horizontal and vertical streaking and smearing and reduced resolution of proteins in 2DE images (for review, Wu et al., 2014b). Therefore, an extensive wash step is usually included to remove such secondary metabolites from adult, recalcitrant plant tissues using acetone/TCA precipitation and phenol extraction. Thus, in the case of leaf midribs from adult maize plants, it is appropriate to use protein extraction protocols based on acetone/TCA precipitation and/or phenol extraction.

### Performance of the three methods in the protein extraction of maize leaf midribs

Proteomic analysis of recalcitrant tissues constitutes an interesting new field with regard to revealing mechanisms related to plant development and responses to environmental cues. Vascular bundles of maize were mostly collected in the midrib that stretches from the lamina joint to the leaf blade. Give the difficulties associated with protein extraction of plant tissues rich in sclerenchyma and vascular bundles, several methods have been used to collect proteins from the xylem and phloem of *Brassica napus* (Kehr et al., 2005), tomato stems (Houterman et al., 2007) and soybean roots (Subramanian et al., 2009). Proteins exhibiting a variety of biological functions (e.g., plant-fungus interactions, xylem-colonization, and deacclimation) were identified and characterized (Abeysekara and Bhattacharyya, 2014). Proteomic analyses of plant recalcitrant tissues constitutes a new field by which mechanisms related to plant development and responses to environmental clues can be illuminated. In maize, 85 xylem sap proteins have been identified that are related to cell wall metabolism, secondary cell wall synthesis, programmed cell death, and plant defense mechanisms (Alvarez et al., 2006, 2008). We have applied TCA/acetone extraction to maize leaf midribs and identified several glycometabolism related proteins (Wang et al., 2015).

We obtained relatively low protein yields in maize leaf midribs (0.93–1.86 mg/g fresh weight, Wang et al., 2015) compared to olive (1.75–2.5 mg/g, Wang et al., 2003) and grape leaves (1.45–3.23 mg/g, Jellouli et al., 2010). In the present study, TCA/acetone extraction produced higher protein yields from maize leaf midribs than the other two methods investigated. Higher protein yields have been reported with TCA/acetone precipitation compared with phenol extraction for several lignified tissues, including leaves and roots (Saravanan and Rose, 2004; Carpentier et al., 2005). The reaction between lignin and Coomassie brilliant blue may provide an explanation for the false positive results obtained via Bradford assays (Sebastiana et al., 2013). Although TCA/acetone extraction produced greater protein yields from finely powdered leaf midribs, washing the powder with 10% TCA/acetone may cause proteins loss due to the precipitation of some proteins in the washing solution. In addition, significant differences exist between the protein patterns obtained via phenol extraction vs. TCA/acetone/phenol extraction. The differences between both extractions occur as a result of the extra washing steps (using 10% TCA/acetone and 80% acetone) and the homogenizing step (using Tris-HCl buffer). A tendency to lose large proteins (e.g., spots 1 and 7) was associated with TCA/acetone/phenol extraction. Fructose-bisphosphate aldolase (spot 7) was only detected in extracts obtained via phenol extraction (Figure 3). Additional steps, such as precipitation and washing steps, may explain the loss of small peptides (Sheoran et al., 2009) that occurs during phenol extraction and TCA/acetone/phenol extraction. Moreover, similar amounts of protein spots have been extracted from leaf midribs via both TCA/acetone and TCA/acetone/phenol extraction methods, indicating that high phenol extraction efficiencies were obtained from the air-dried midrib powder. The performances of the three extraction methods evaluated herein indicate that, although TCA/acetone extraction produced high protein yields, interfering substances and washing steps may affect Bradford assays or cause losses of proteins with high molecular masses. Phenol extraction was superior with regard to avoiding these problems due to benefits associated with the separation of proteins and interfering substances due to the use of buffered phenol prior to methanol/ammonium acetate precipitation and acetone washing.

In the present study, 16 differentially extracted proteins from maize leaf midribs among the three extraction methods were identified as hydrophilic proteins. Hydrophilic proteins located within chloroplasts demonstrated to be important fractions with regard to plant proteomics. Recently, several studies analyzed the entire chloroplast proteome in maize and Arabidopsis by 2DE using different protein extraction procedures (i.e., TCA/acetone precipitation and acetone precipitation) (Lonosky et al., 2004; Phee et al., 2004). In the present study, three chloroplast proteins, chlorophyll a-b binding protein 8 (spot 12), cytochrome b6-f complex iron-sulfur subunit (spot 16) and chlorophyll a-b binding protein 6A (spot 17), were detected and identified via TCA/acetone-based extraction. By contrast, the three thylakoid or chloroplast-membrane proteins were lost during phenol extraction. Previous studies demonstrated the difficulties associated with plant membrane proteins due to their low solubility in the rehydration buffers and electrophoretic properties in IEF (Santoni et al., 2000). Spot numbers from 2DE followed by MS identification were lower than those obtained from direct identification using gel-free strategies, especially for membrane proteins, as previously reported in rice and Arabidopsis (Marmagne et al., 2004; Tanaka et al., 2004). Furthermore, these results demonstrate the intrinsic limitations of the extractions evaluated herein in determining total chloroplast proteins, particularly given the insufficient extraction of hydrophobic membrane proteins in the envelope and the thylakoid. A major difference among the three extraction methods was that the initial treatment applied to the powdered leaf material, i.e., the phenol method involved a Tris buffer extraction of hydrophilic proteins, whereas the other two methods used TCA/acetone precipitation which is based on protein denaturizing under acidic and/or hydrophobic conditions that help to concentrate proteins and remove contaminants (Damerval et al., 1986). Therefore, for studies targeting hydrophobic proteins, it would be possible to modify the phenol extraction protocol presented here, to include detergents or chaotropes that would facilitate solubilization of hydrophobic proteins.

In summary, all three of the methods evaluated herein enabled us to obtain high-quality protein samples and good 2DE maps for maize leaf midrib, although phenol extraction produced a better 2DE map with more resolved spots, and TCA/acetone extraction produced higher protein yields. In addition, due to the protein extraction preferences (major hydrophilic proteins) of these three extraction methods, extraction of hydrophobic proteins is optimal for proteome profiling of maize leaf midribs. The present study provides useful information with regard to selecting suitable protein extraction methodology or modulating these protocols for proteome analysis of recalcitrant plant tissues rich in sclerenchyma cells.

## Author contributions

NW performed the experiments, analyzed the data and drafted the manuscript. XW assisted in experiments and data analysis and edited the manuscript. LK and YC provided materials. WW edited the manuscript and supported the study.

### Conflict of interest statement

The authors declare that the research was conducted in the absence of any commercial or financial relationships that could be construed as a potential conflict of interest.
