# Supplementary material for: Evaluation of Three Protein-Extraction Methods for Proteome Analysis of Maize Leaf Midrib, a Compound Tissue Rich in Sclerenchyma Cells
Source: Front Plant Sci. 2016 Jun 14;7:856. doi: 10.3389/fpls.2016.00856 (PMC4905967; doi:10.3389/fpls.2016.00856)
Supplement: Supplementary file 1 [file Table1.DOC]

**Supplementary Table S1****∣** MS/MS identification of differential proteins among the three protein-extraction methods in maize leaf midribs.

| **Spot** | **NCBI accession** | **UniProtKB accession** | **Protein** | **Mr/p*I*** | **Coverage (%)** | **Mascot score** | **Matched sequences** |
| --- | --- | --- | --- | --- | --- | --- | --- |
| 1 | 227786 | P00827 | ATP synthase subunit beta | 54.10/5.31 | 30 | 1068 | R.GMEVIDTGTPLSVPVGGATLGR.I, R.IFNVLGEPIDNLGPVDTSATFPIHR.S, K.AHGGVSVFGGVGER.T, K.VALVYGQMNEPPGAR.M, R.VGLTALTMAEYFR.D, R.VGLTALTMAEYFR.D + Oxidation (M), R.DVNKQDVLLFIDNIFR.F, R.FVQAGSEVSALLGR.M, K.GIYPAVDPLDSTSTMLQPR.I, R.IVGNEHYETAQR.V |
| 2 | 227786 | P00827 | ATP synthase subunit beta | 54.10/5.31 | 30 | 1068 | R.GMEVIDTGTPLSVPVGGATLGR.I, R.IFNVLGEPIDNLGPVDTSATFPIHR.S, K.AHGGVSVFGGVGER.T, K.VALVYGQMNEPPGAR.M, R.VGLTALTMAEYFR.D, R.VGLTALTMAEYFR.D + Oxidation (M), R.DVNKQDVLLFIDNIFR.F, R.FVQAGSEVSALLGR.M, K.GIYPAVDPLDSTSTMLQPR.I, R.IVGNEHYETAQR.V |
| 3 | [131998](http://www.matrixscience.com/cgi/master_results.pl?file=..%2Fdata%2F20150828%2FFTTAlaEae.dat;sessionID=guest_guestsession#Hit1) | P19163 | RuBisCO large subunit; Flags: Precursor | 53.54/6.09 | 18 | 593 | R.ACYECLR.G, K.DTDILAAFR.V, R.FVFCAEAIYK.A, R.EITLGFVDLLR.D, K.LTYYTPEYETK.D, R.DNGLLLHIHR.A, R.GGLDFTKDDENVNSQPFMR.W, R.VALEACVQAR.N |
| 4 | [226508814](http://www.matrixscience.com/cgi/master_results.pl?file=..%2Fdata%2F20150828%2FFTTAlaYTS.dat;sessionID=guest_guestsession#Hit1) | B6T9J4 | Aspartate aminotransferase | 50.55/8.15 | 21 | 501 | K.LNLGVGAYR.T, R.TEELQPYVLNVVK.K, K.EYLPIEGLAAFNK.A, K.VLISSPTWGNHK.N + Dioxidation (W), R.VPWSEYR.Y, R.GMEVFVAQSYSK.N, K.NLGLYSER.V, R.VGAINVVCSAPEVADR.V, K.DWSFILR.Q + Dioxidation (W) |
| 5 | [223975775](http://www.matrixscience.com/cgi/master_results.pl?file=..%2Fdata%2F20150828%2FFTTAlaYnm.dat;sessionID=guest_guestsession#Hit1) | C0PD30 | Fructose-bisphosphate aldolase | 38.41/6.37 | 36 | 820 | R.LASIGLENTEANR.Q, R.TLLVTAPGLGQYISGAILFEETLYQSAVDGR.K, K.AAQDALLLR.A, K.GLVPLAGSNNESWCQGLDGLASR.E,R.EAAYYQQGAR.F, K.IVDILAEQGIVPGIK.V, R.YAAISQDNGLVPIVEPEILLDGEHGIER.T |
| 6 | [22240](http://www.matrixscience.com/cgi/master_results.pl?file=..%2Fdata%2F20150828%2FFTTAlaSOT.dat;sessionID=guest_guestsession#Hit1) | Q6LBU9 | Glyceraldehyde-3-phosphate dehydrogenase | 41.27/7.21 | 29 | 821 | R.GDASPLDVIAINDTGGVK.Q, K.GTMTTTHSYTGDQR.L, K.GTMTTTHSYTGDQR.L + Oxidation (M), R.VPTPNVSVVDLVVQVSK.K, K.KTLAEEVNQAFR.D, K.TLAEEVNQAFR.D, R.VVDLADICANQWK.- R.DAAANELTGILEVCDVPLVSVDFR.C, K.VISWYDNEWGYSQR.V, K.VISWYDNEWGYSQR.V + Dioxidation (W) |
| 7 | [223975775](http://www.matrixscience.com/cgi/master_results.pl?file=..%2Fdata%2F20150828%2FFTTAlaYnm.dat;sessionID=guest_guestsession#Hit1) | C0PD30 | Fructose-bisphosphate aldolase | 38.41/6.37 | 36 | 820 | R.LASIGLENTEANR.Q, R.TLLVTAPGLGQYISGAILFEETLYQSAVDGR.K, K.IVDILAEQGIVPGIK.V, K.GLVPLAGSNNESWCQGLDGLASR.E, R.EAAYYQQGAR.F, R.YAAISQDNGLVPIVEPEILLDGEHGIER.T, K.AAQDALLLR.A |
| 8 | [223975775](http://www.matrixscience.com/cgi/master_results.pl?file=..%2Fdata%2F20150828%2FFTTAlaYnm.dat;sessionID=guest_guestsession#Hit1) | C0PD30 | Fructose-bisphosphate aldolase | 38.41/6.37 | 36 | 820 | R.LASIGLENTEANR.Q, R.TLLVTAPGLGQYISGAILFEETLYQSAVDGR.K, K.IVDILAEQGIVPGIK.V, K.GLVPLAGSNNESWCQGLDGLASR.E, R.EAAYYQQGAR.F, R.YAAISQDNGLVPIVEPEILLDGEHGIER.T, K.AAQDALLLR.A |
| 9 | [670412710](http://www.matrixscience.com/cgi/master_results.pl?file=..%2Fdata%2F20150828%2FFTTAlaSnO.dat;sessionID=guest_guestsession#Hit1) | B6T3B2 | Oxygen-evolving enhancer protein 1 | 34.78/5.59 | 39 | 975 | K.RLTYDEIQSK.T, K.GTGTANQCPTIDGGVESFPFK.A, K.NAPPEFQK.T, K.DGIDYAAVTVQLPGGER.V, K.FEEKDGIDYAAVTVQLPGGER.V, K.QLVATGKPESFGGPFLVPSYR.G, R.GGSTGYDNAVALPAGGR.G, K.SNPETGEVIGVFESVQPSDTDLGAK.A, R.VPFLFTVK.Q, |
| 10 | [670412710](http://www.matrixscience.com/cgi/master_results.pl?file=..%2Fdata%2F20150828%2FFTTAlaSnO.dat;sessionID=guest_guestsession#Hit1) | B6T3B2 | Oxygen-evolving enhancer protein 1 | 34.78/5.59 | 39 | 975 | K.RLTYDEIQSK.T, K.GTGTANQCPTIDGGVESFPFK.A, K.NAPPEFQK.T, K.DGIDYAAVTVQLPGGER.V, K.FEEKDGIDYAAVTVQLPGGER.V, K.QLVATGKPESFGGPFLVPSYR.G, R.GGSTGYDNAVALPAGGR.G, K.SNPETGEVIGVFESVQPSDTDLGAK.A, R.VPFLFTVK.Q, |
| 11 | [195642948](http://www.matrixscience.com/cgi/master_results.pl?file=..%2Fdata%2F20150828%2FFTTAlaSnS.dat;sessionID=guest_guestsession#Hit1) | B6TV09 | 3-beta hydroxysteroid dehydrogenase/isomerase family protein | 32.77/7.63 | 13 | 175 | K.IVVLGGSGFVGSAICR.A, K.GIEVVSFSR.S, K.YPASGVVLRPGFIYGK.R |
| 12 | [413954857](http://www.matrixscience.com/cgi/master_results.pl?file=..%2Fdata%2F20150828%2FFTTAlaSEe.dat;sessionID=guest_guestsession#Hit1) | K7VFU9 | Chlorophyll a-b binding protein 8 | 22.96/5.79 | 41 | 563 | K.WLAYGEVINGR.Y, R.YAMLGAVGAIAPEIFGK.M + Oxidation (M), K.MGIIPPETALPWFK.T + Dioxidation (W); Oxidation (M), R.RLQDWYNPGSMGK.Q + Oxidation (M), R.RLQDWYNPGSMGK.Q + Dioxidation (W); Oxidation (M), R.LQDWYNPGSMGK.Q, R.LQDWYNPGSMGK.Q + Oxidation (M), K.QYFLGLEK.F, K.FLAGSGDPSYPGGPLFNPLGFGK.T |
| 13 | [226505920](http://www.matrixscience.com/cgi/master_results.pl?file=..%2Fdata%2F20150828%2FFTTAlaSeO.dat;sessionID=guest_guestsession#Hit1) | B4FT31 | Dehydroascorbate reductase | 23.51/5/54 | 30 | 391 | K.AAAGNPDTLGDCPFSQR.V, K.LVDLGNKPEWFLNISPEGK.V + Dioxidation (W), K.ALLDELQALDDHLK.A, K.TKPSEEHVIAGWAPK.V |
| 14 | [162458009](http://www.matrixscience.com/cgi/master_results.pl?file=..%2Fdata%2F20150828%2FFTTAlaSeR.dat;sessionID=guest_guestsession#Hit1) | P80639 | Eukaryotic translation initiation factor 5A | 17.71/5.61 | 57 | 663 | M.SDSEEHHFESK.A + Acetyl (Protein N-term), K.TYPQQAGTVR.K, K.CHFVAIDIFNGK.K + Deamidated (NQ), K.KLEDIVPSSHNCDIPHVNR.T, K.LEDIVPSSHNCDIPHVNR.T, R.TEYQLIDISEDGFVSLLTSDGNTK.D, K.DDLRLPTDETLVAQIK.E, R.LPTDETLVAQIK.E |
| 15 | [226491656](http://www.matrixscience.com/cgi/master_results.pl?file=..%2Fdata%2F20150828%2FFTTAlaSmh.dat;sessionID=guest_guestsession#Hit1) | B6TCE9 | Peptidyl-prolyl cis-trans isomerase | 26.37/9.28 | 33 | 608 | K.VYFDISIGNPVGK.N, R.IVIGLYGDDVPQTTENFR.A, R.VISDFMIQGGDFDK.G + Oxidation (M), R.VISDFMIQGGDFDKGNGTGGK.S + Deamidated (NQ), R.VISDFMIQGGDFDKGNGTGGK.S + Deamidated (NQ); Oxidation (M), R.TFKDENFK.L, R.HVVFGQVLEGMDVVSLIESQETDR.G + Oxidation (M) |
| 16 | 194702912 | B4FTU7 | Cytochrome b6-f complex iron-sulfur subunit | 21.03/6.41 | 44 | 680 | K.DKLGNDITVEAWLNTHGPNDR.T + Dioxidation (W), R.TGEDPWWK.A, K.LGNDITVEAWLNTHGPNDR.T + Dioxidation (W), K.GDPTYLVVEQDK.T, K.FICPCHGSQYNNQGK.V, R.GPAPLSLALVHADVDDGK.V, K.VLFVPWVETDFR.T + Dioxidation (W), |
| 17 | 194702912 | B4FTU7 | Cytochrome b6-f complex iron-sulfur subunit | 21.03/6.41 | 44 | 680 | K.DKLGNDITVEAWLNTHGPNDR.T + Dioxidation (W), R.TGEDPWWK.A, K.LGNDITVEAWLNTHGPNDR.T + Dioxidation (W), K.GDPTYLVVEQDK.T, K.FICPCHGSQYNNQGK.V, R.GPAPLSLALVHADVDDGK.V, K.VLFVPWVETDFR.T + Dioxidation (W), |
| 18 | [226530077](http://www.matrixscience.com/cgi/master_results.pl?file=..%2Fdata%2F20150828%2FFTTAlaSwe.dat;sessionID=guest_guestsession#Hit2) | B4FZL4 | Chlorophyll a-b binding protein 6A | 26.83/6.30 | 9 | 165 | R.FKESEVYHCR.W, K.ESEVYHCR.W, K.YPGGAFDPLGFSR.D |

Spots exhibiting 2-fold abundance changes between any two of the three protocols were identified as differential proteins and were digested for MS/MS analysis.
