# Supplementary figures and images for: Evaluation of Three Protein-Extraction Methods for Proteome Analysis of Maize Leaf Midrib, a Compound Tissue Rich in Sclerenchyma Cells
Source: Front Plant Sci. 2016 Jun 14;7:856. doi: 10.3389/fpls.2016.00856 (PMC4905967; doi:10.3389/fpls.2016.00856)

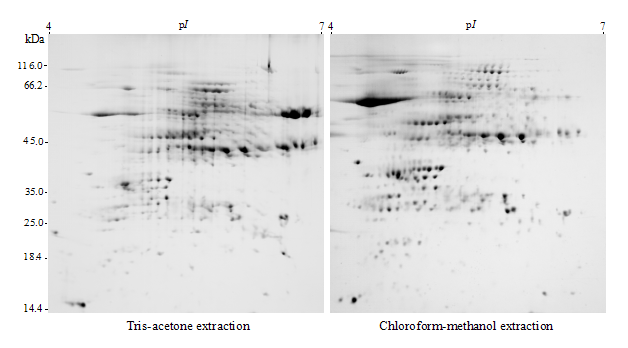

Supplement: Supplementary file 2 [file Image1.TIF]
